# Opinion Leaders and Structural Hole Spanners Influencing Echo Chambers in Discussions About COVID-19 Vaccines on Social Media in China: Network Analysis

**DOI:** 10.2196/40701

**Published:** 2022-11-18

**Authors:** Dandan Wang, Yadong Zhou, Feicheng Ma

**Affiliations:** 1 School of Information Management Wuhan University Wuhan China; 2 School of Data Science City University of Hong Kong Hong Kong China (Hong Kong); 3 Center for Studies of Information Resources Wuhan University Wuhan China; 4 Big Data Institute Wuhan University Wuhan China; 5 Department of Electrical and Computer Engineering University of Florida Gainesville, FL United States

**Keywords:** COVID-19, COVID-19 vaccine, echo chamber, opinion leader, structural hole spanner, topic, sentiment, social media, vaccine hesitancy, public health, vaccination, health promotion, online campaign, social network analysis

## Abstract

**Background:**

Social media provide an ideal medium for breeding and reinforcing vaccine hesitancy, especially during public health emergencies. Algorithmic recommendation–based technology along with users’ selective exposure and group pressure lead to online echo chambers, causing inefficiency in vaccination promotion. Avoiding or breaking echo chambers largely relies on key users’ behavior.

**Objective:**

With the ultimate goal of eliminating the impact of echo chambers related to vaccine hesitancy on social media during public health emergencies, the aim of this study was to develop a framework to quantify the echo chamber effect in users’ topic selection and attitude contagion about COVID-19 vaccines or vaccinations; detect online opinion leaders and structural hole spanners based on network attributes; and explore the relationships of their behavior patterns and network locations, as well as the relationships of network locations and impact on topic-based and attitude-based echo chambers.

**Methods:**

We called the Sina Weibo application programming interface to crawl tweets related to the COVID-19 vaccine or vaccination and user information on Weibo, a Chinese social media platform. Adopting social network analysis, we examined the low echo chamber effect based on topics in representational networks of information, according to attitude in communication flow networks of users under different interactive mechanisms (retweeting, commenting). Statistical and visual analyses were used to characterize behavior patterns of key users (opinion leaders, structural hole spanners), and to explore their function in avoiding or breaking topic-based and attitude-based echo chambers.

**Results:**

Users showed a low echo chamber effect in vaccine-related topic selection and attitude interaction. For the former, the homophily was more obvious in retweeting than in commenting, whereas the opposite trend was found for the latter. Speakers, replicators, and monologists tended to be opinion leaders, whereas common users, retweeters, and networkers tended to be structural hole spanners. Both leaders and spanners tended to be “bridgers” to disseminate diverse topics and communicate with users holding cross-cutting attitudes toward COVID-19 vaccines. Moreover, users who tended to echo a single topic could bridge multiple attitudes, while users who focused on diverse topics also tended to serve as bridgers for different attitudes.

**Conclusions:**

This study not only revealed a low echo chamber effect in vaccine hesitancy, but further elucidated the underlying reasons from the perspective of users, offering insights for research about the form, degree, and formation of echo chambers, along with depolarization, social capital, stakeholder theory, user portraits, dissemination pattern of topic, and sentiment. Therefore, this work can help to provide strategies for public health and public opinion managers to cooperate toward avoiding or correcting echo chamber chaos and effectively promoting online vaccine campaigns.

## Introduction

### Background

Despite scientific consensus that COVID-19 vaccines are safe and effective [1], there is still widely circulated controversial information on social media, with statements such as “while vaccinations offer good protection, they do not provide full immunity, and the extent to which they would be effective against new variants of the virus remains uncertain,” which damages public confidence [2]. This misinformation leads to vaccine hesitancy, which has been recognized by the World Health Organization as a major global health threat [3]. Social media platforms such as Twitter, Facebook, TikTok, and YouTube provide an ideal medium for spreading and reinforcing antivaccine ideas [4-7]. First, the information-filtering mechanism based on algorithmic recommendation technology mediates and facilitates content promotion by considering users’ interest and attitudes [8]. Second, online users have access to a wealth of information and narratives. Affected by individual and social factors such as selective exposure and group pressure, users prefer to select information that fits their belief system, while ignoring dissident information. Gradually, echo chambers emerge, in which like-minded people continue to frame and strengthen shared narratives [9]. In the vaccine promotion campaign, the trend of simplification of users’ vaccine-information sources is strengthened and the flow of information between groups with different ideologies toward vaccines is blocked, which widens the knowledge gap and assimilates value cognition [10]. The accompanying group polarization and social fragmentation blind the public to preconceived misconceptions and undermine authorities’ efforts to improve the public’s information literacy [11], causing inefficiency in the vaccine campaign [12,13].

Users in a social network can be divided into three roles: opinion leaders, structural hole spanners, and ordinary users [14]. Lou and Tang [15] pointed out that the top 1% of users acting as structural hole spanners control almost 80% of information diffusion between communities and 25% of information diffusion on Twitter. Wu et al [16] revealed that 50% of URLs were posted by 1% of users serving as opinion leaders. Further, Cossard et al [17] identified key users in echo chambers, while Jeon et al [18] evaluated the characteristics of users who broke the echo chamber. 

To avoid or break an echo chamber, it is critical to characterize these key users and determine their impact on topic dissemination and opinion evolution, which could facilitate the communication within and between pro- and antivaccine groups, and thereby eliminate vaccine hesitancy. Toward this end, in this study, we developed a framework to evaluate and compare the degree of the effect of different forms of echo chambers on users’ interactive behavior using quantitative measurements. We further explored the hidden mechanisms of an echo chamber’s formation and its strengthening or disintegration by detecting key users who occupy critical network positions, analyzing the relationship between their behavior pattern, network location, and function both inside and outside of echo chambers. Although this framework was designed based on online debates of COVID-19 vaccine hesitancy as the background to offer insights for public health administrators, it could also be applied and expanded to other controversial theme discussions to serve as a reference for public opinion managers.

### Prior Work

#### Echo Chamber of Vaccine Hesitancy

Most studies in this field have concentrated on the presence, form, and degree of echo chambers, whereas limited research has aimed to develop efficient strategies to address the echo chamber effect. Schmidt et al [6]analyzed vaccine-related posts on Facebook from 2010 to 2017, claiming the existence of highly polarized pro- and antivaccine groups by calculating each user’s attitude-polarization score based on their “like” and comment behavior. Mønsted and Lehmann [5] obtained similar results from an analysis of tweets posted on Twitter from 2013 to 2016, using the assortativity coefficient derived from network structures. Rathje et al [19] adopted the same index to examine the degree of the echo chamber effect during the COVID-19 epidemic. Apart from attitude-based self-isolation, Del Vicario et al [20] found highly controversial topics by measuring the distance between how a certain topic is presented in tweets and the related users’ emotional response. Cossard et al [17] further compared the echo chamber effect on users’ interactive behaviors (retweeting, mentioning) on Twitter during measles outbreaks, and identified key users occupying a central location in interactive networks to tighten the structures of echo chambers. To mitigate the negative effect of echo chambers, Jeon et al [18] performed a user experiment using a game-based methodology to determine the characteristics of users who broke the echo chamber. The breakers were consistently aware of being trapped in echo chambers and tended to maintain diverse perspectives when consuming information.

#### User Roles in Echo Chambers

Social capital, as a set of resources embedded in relationships, results from holding certain locations in a social structure [21]. Social capital theory suggests that a more central location in a social network, with cohesive social ties fostering trust and cooperation, leads to more bonding relationships. By contrast, structural hole theory emphasizes that social capital results from a bridging position, which can bring the ego diverse and nonredundant information [22,23], as well as control of information flow [24] so as to enhance innovation performance [25]. The idea is grounded in weak tie theory [26]. Weak ties represent loose connections in the network, making it easier to include a large number of talents with different views, information, and resources [27].

Burt [28] explained that whether social capital performs a greater function of bonding than bridging depends on the context. An “opinion leader” is a term used to broadly refer to any individual or entity with high influence in a network, and should not be predetermined but rather explored in different contexts [29]. Opinion leaders occupy the center of the information network within their local communities, and can influence others by drawing their attention to certain topics or opinions and inspiring reactions to the messages they post [30-32]. Opinion leaders have been found to be responsible for promoting an echo chamber [33]. Through an online-search experiment, Bar-Gill and Gandal [34] found that opinion leaders raised the potential for a topic echo chamber, promoting communities to focus on homogeneous topics. Guo et al [29] analyzed the impact of opinion leaders of different genders, partisanship, and stakeholder categories on political homophily in Twitter communities. However, Dubois and Blank [35] and Dubois et al [36] drew conclusions from survey data that the contribution of opinion leaders to a political echo chamber was overvalued without considering the interests of information receivers and the diversity of information sources. Based on thorough qualitative interviews, Bergström and Jervelycke Belfrage [37] also argued that opinion leaders brought attention to news others would have missed.

The lack of connection among communities forms structural holes in social structures [38]. Individuals filling the holes, acting as intermediaries between different communities, are regarded as “structural hole spanners” [15,39]. By simulating opinion update rules of ordinary agents and structural hole agents, Gong et al [40] proved that structural hole–based approaches could alleviate the echo chamber effect and reduce opinion polarity in social networks. Using social network analysis, Swarnakar et al [41] emphasized that structural hole spanners acted as brokers and bridge-makers for collaboration of heterogeneous patterns on climate change.

Research about opinion leaders’ impact on echo chambers has resulted in contradictory conclusions with respect to different social issues. Limited research has focused on the impact of structural hole spanners on echo chambers. Rather, research in this field has mainly focused on opinion-based echo chambers under specific topics, while ignoring users’ topic selection prior to opinion contagion. Despite these advancements, a gap remains in the literature: if both bonding and bridging arguments are valid depending on the context, under which conditions should they be complementary or otherwise?

### Research Questions

Online echo chambers have been studied in the context of users’ interactions (eg, posting, retweeting, commenting, mentioning), focusing on rumor spread and management [42-44], political debates [29,45], and news consumption [46,47]; however, related studies on vaccine hesitancy are rare, especially during public health emergencies. To assess whether users on Sina Weibo, the most popular microblogging platform in China (with a structure similar to Twitter), exhibited an echo chamber effect when discussing COVID-19 vaccines and vaccinations, and to further understand the formation mechanism or to design strategies to break it, we sought to identify the key users, how their behavior patterns relate to their online positions, and how they cooperate or compete to promote (prevent) the formation or strengthening (breaking) of the echo chamber. Toward this end, we established the following research questions (RQs):

RQ1: Is there an echo chamber effect in topic selection and opinion contagion of users on Weibo when discussing COVID-19 vaccines and vaccinations? Does it differ between users’ retweeting and commenting behaviors?

RQ2: Do users with different behavior patterns on Weibo tend to be regarded as opinion leaders or structural hole spanners?

RQ3a: Do online opinion leaders and structural hole spanners tend to act as echoers or bridgers in topic dissemination?

RQ3b: Do online opinion leaders and structural hole spanners tend to act as echoers or bridgers in attitude interaction?

RQ4a: Do these key users acting as echoers in topic dissemination tend to play the same role in attitude interaction?

RQ4b**:** Do these key users acting as bridgers in topic dissemination tend to play the same role in attitude interaction?

## Methods

### Design and Definitions

**Figure 1:**
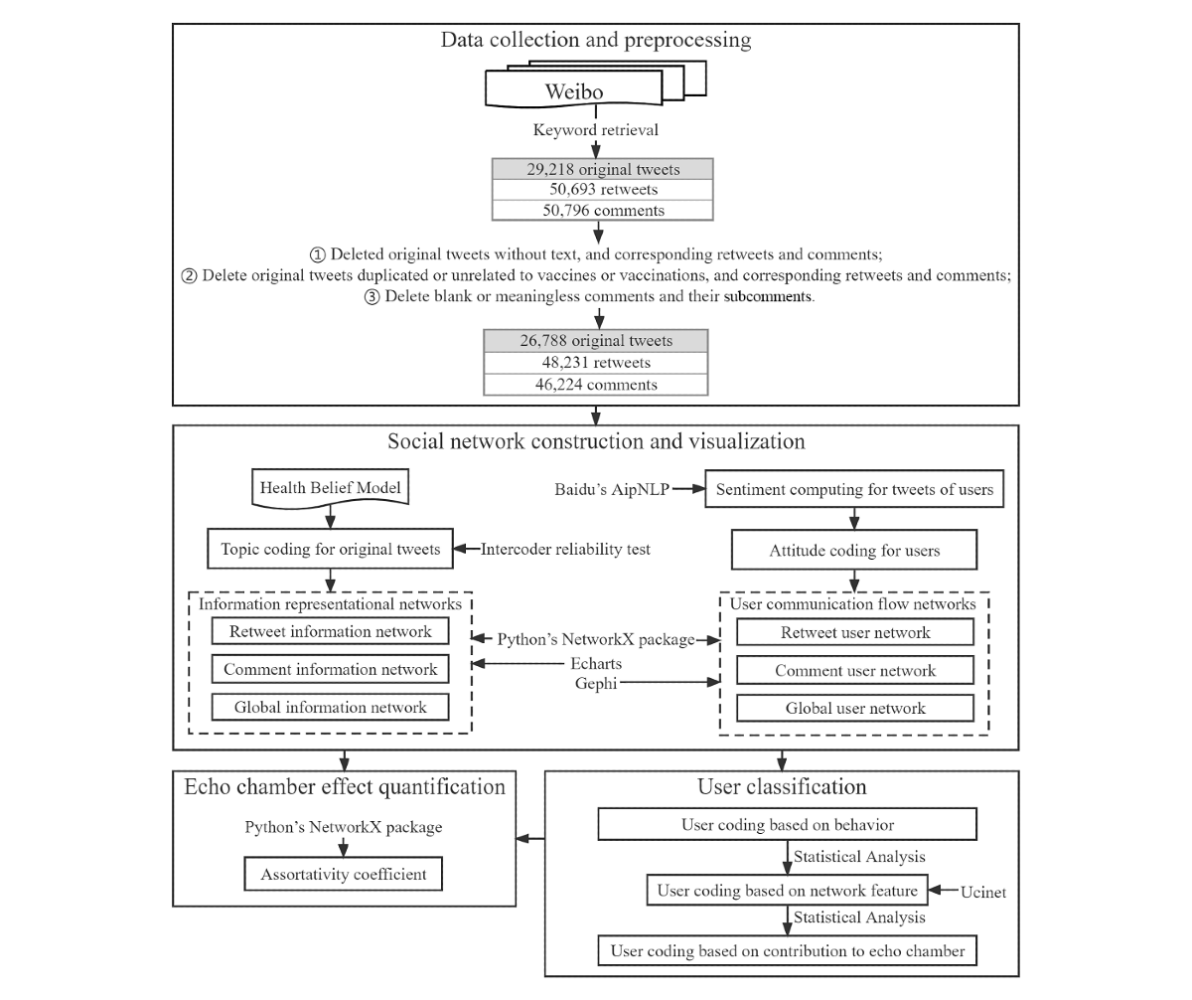
Research framework.

Figure 1 outlines the research framework. Note that although Weibo posts were analyzed in this study, we use the terms “tweet” and “retweet” throughout the manuscript to refer to activities on the platform, equivalent to activities on Twitter, for the sake of convenience. An original “tweet” refers to posts created by a registered Weibo user. A “retweet” refers to users’ forwarding behavior on Weibo. “Comments” refer to replies to an original post on Weibo.

### Ethical Considerations

Our research did not require ethical board approval because it did not involve human or animal trials. The research data were derived from open access data available on social media, mainly through voluntary contributions from users. Our data and analysis of data were conducted in an unbiased and transparent manner, and the data were used only for scientific research without any ethical violations. To be specific, we anonymized key identifiable information, including the nickname field provided by each user and the ID number assigned to each user by the platform when they registered their unique account. We represented these two fields as nonrepeating consecutive integers incremented from 1 to uniquely identify each user, thus hiding the users’ personal information, which had no influence on the study results.

### Data Collection and Preprocessing

From January 23, 2020, to February 11, 2021, there were numerous messages posted about the outbreak and cessation of the COVID-19 epidemic, as well as the initial exploration of vaccine development and vaccination on Weibo [48]. As a medical innovation, the vaccine was widely debated in its early diffusion stage [49]. We first used the Sina Weibo application programming interface to collect original tweets containing keywords (“COVID-19 vaccine [新冠疫苗]” or “COVID-19 vaccination [新冠疫苗接种]”). Considering that the interactive data (ie, retweet, comment, and like) of an original tweet could become stable approximately 1 week after it was posted [50,51], we crawled the following-week interactive data for each original tweet, involving likes, retweets, and comments, and the information of posters, retweeting, and commenting users. There were initially 29,218 original tweets, 50,693 retweets, and 50,796 comments.

For data preprocessing, we deleted the original tweets without any text (eg, only pictures, videos, or audio) or those that were duplicated or contained the above keywords but did not include any meaningful content. In addition, blank or meaningless comments and their subcomments were also eliminated. After excluding the corresponding retweets and comments as well as the user information, there were 26,788 original tweets, 48,231 retweets, and 46,224 comments from 77,625 users retained for analysis.

### Social Network Construction and Visualization

#### Interactive Network Design

To answer RQ1-4, we constructed interactive networks. Information representational and user communication flow networks are commonly used as the basis to measure polarization [20,43,44].

#### Information Representational Network Construction

First, we marked the topic for each original tweet. To cover all aspects of the vaccine, we performed this process based on the Health Belief Model, which indicates that the perceived susceptibility, perceived severity, perceived benefits, perceived barriers, cues to action, and self-efficacy have impacts on individuals’ motivation to carry out preventive health behaviors [52,53]. We invited two experienced researchers to label the topics for 10% of the original tweets, and the result passed intercoder reliability tests [54] (κ=0.967). After repeating the review and eliminating disagreements, the topic-coding scheme was developed (we did not consider the construct of self-efficacy owing to its low prevalence in the data set), which is shown in Table 1. This scheme was used to label the remaining original tweets.

Retweeting or commenting on an original tweet indicates that the users are interested in the tweet’s topic [20,44]. Based on interactive data, we next established representational networks of information, which were undirected and weighted. In a global information network, each node represents an original tweet; if a user retweets or comments on original tweet *i* and original tweet *j*, an edge exists between *i* and *j*. The edge’s weight represents the number of common users who participate in discussion on the two original tweets. The retweet/comment information network only contained retweet/comment relationships. We then used Python’s NetworkX package to construct these three networks [55] and calculated their detailed topological attributes. Finally, the chord diagram visualization of Echarts [56] was used to visualize the degree of homophily based on topics in the networks.

**Table 1:** Topic-coding scheme based on the Health Belief Model.

Construct	Topics
Perceived susceptibility	Risk of getting COVID-19 infection
Perceived severity	Severity of getting COVID-19 infection or refusing COVID-19 vaccination
Perceived benefits	Effectiveness of COVID-19 vaccination
Perceived barriers	Adverse effects of COVID-19 vaccination; cost of COVID-19 vaccination; fake (eg, counterfeit) vaccines, fraudulent information; safety (eg novelty), infectivity of vaccines, and standardization of vaccination process; conspiracy theory
Cues to action	Means to get vaccination; dos and don’ts of vaccination; domestic vaccine development, production, and vaccination; foreign vaccine development, production, and vaccination; personal vaccination experience

#### User Communication Flow Network Construction

Many studies adopted the sentiment expressed in tweets created/retweeted/commented by users to represent their attitudes toward vaccines [5,20,57]. Considering the Chinese context of Weibo [58], we used Baidu’s AipNLP [59] to calculate the sentimental positive probability (0≤α≤1) of each original tweet, retweet, and comment. If 0≤α≤0.5, the text was regarded as negative; if 0<α≤1, the text was regard as positive; and otherwise, it was regard as neutral. We counted the most frequently expressed sentiment type of the user, which represented their attitude toward vaccines. Next, we established communication flow networks of users, which were directed and weighted. In the global user network, each node represents a user; if user *i* retweets or comments on tweets (including original tweets, retweets, and comments) of user *j*, there is an edge from user *i* to *j*. The edge’s weight represents the number of interactions between the two users. The retweet/comment user network only contained retweet/comment relationships. We then used Python’s NetworkX package to construct these three networks [55] and calculated their detailed topological attributes. Finally, Gephi was used to visualize the degree of homophily based on users’ attitudes, and the Fruchterman Reingold layout algorithm was used to visualize the connectivity in user networks [60].

### Echo Chamber Effect Quantification

To answer RQ1, we used Python’s NetworkX package to calculate each network’s assortativity coefficient *r* (–1≤*r*≤1) based on the nodes’ attributes (topic in information networks, attitude in user networks) and their interaction, which measures the network’s homophily [5,44]. An *r*>0 indicates that the node generally tends to connect with other nodes with similar properties, and the network is referred to as an assortative network. A larger *r* value indicates more prominent assortativity. If *r*≤0, assortativity does not hold [61].

### User Classification

#### Method of Classification

To answer RQ2, we characterized online users’ behavior patterns and detected opinion leaders and structural hole spanners based on their network locations. To answer RQ3-4, we defined two types of mediators to represent the above key users’ contributions to echo chambers. After coding users from these three perspectives, statistical tests were used to examine the relationships.

#### User Coding Based on Behavior

Villodre and Criado [62] classified users based on their contrasting behaviors during the dissemination of crisis information. Based on a modification of their rules, we classified all users into 8 categories, as shown in Table 2. We then analyzed stakeholders for each category by matching keywords in each user’s personal authentication, introduction, and tags. Referring to the identity-keyword list from An and Ou [63], after manually marking 10% of all users (intercoder reliability of two coders, κ=0.991), we modified and expanded the list, and finally determined 11 categories, as shown in Table 3. The remaining users were automatically coded using the new list.

**Table 2:** User behavior taxonomy.

User category		Criterion	Behavior description
**Influential**			
	Speaker	Number of retweets received was three times higher (low speakers), 10 times higher (medium speakers), or 100 times higher (high speakers) than that of tweets they had posted	Users create widely shared content. They show less content-sharing behavior
	Networker	Number of tweets≥total mean; number of retweets received≥total mean; number of retweets received/number of retweets sent≥0.5	Users show equilibrium between creating content, sharing content, and being retransmitted
**Broadcaster**			
	Monologist	Number of tweets≥total mean; number of retweets received/own tweets≤0.3	Users create original content that is not widely shared
	Retweeter	Number of tweets≥total mean; number of retweets sent/own tweets≥0.5	Users mostly share others’ content
	Replicator	Number of comments sent/own tweets≥0.6	Users mostly comment on others’ content
	Isolator	Number of retweets sent=0; number of retweets received=0; number of comments sent=0; number of comments received=0	Users never share/comment on others’ content and they create some content that is never shared/commented by others
	Automatic	Send same comments multiple times under one tweet; personal information is blank	Users seem to act with automatization
Common user		None of the above	Not applicable

**Table 3:** Stakeholder types and related keywords.

Stakeholder types	Keywords (partial)
Government	government, police, court, judicial bureau, judicial office, procuratorate, commission for discipline inspection, political and legal committee
Hospital	hospital
Traditional media	newspaper, radio, TV station, news, magazine, broadcast, daily, timely, weekly, monthly, morning post, evening post, channel
We-media	We-media, author, writer, reporter, editor, blogger, commentator, critic
Platform account	Sina Weibo, Weibo medical and health operation, Weibo secretary, Weibo administrator, Weibo rumor rebuttal, Weibo politics
Social organization	association, public welfare
Medical company	vaccine manufacturer (“SINOVAC BIOTECH CO., LTD”. [“科兴”], “CanSino Biologics Inc.” [“康希诺”], “Hualan,” “Zhifei,” “Kangtai”), medical enterprise
Common company	company, enterprise
Educational institution	middle school, high school, campus, technical school
Medical personnel	doctor, nurse
Common personnel	None of the above

#### User Coding Based on Network Features

To measure the extent of each user being regarded as an opinion leader, we adopted in-degree centrality [29], which represents the volume of network ties directed toward a user [64], and the local clustering coefficient, which quantifies the degree to which the user’s neighbors aggregate with each other to form a clique (complete graph) [65]. Burt [38] proposed four metrics to describe structural hole spanners: effective size, efficiency, constraint, and hierarchy, the third of which is the most important. The effective size of a node measures the nonredundant connections of a node. Efficiency is the effective size divided by the number of the node’s neighbors. Yang et al [25] and Tan et al [66] chose “constraint” (between 0 and 1), which measures the extent to which the node’s contacts are redundant. When the constraint is closer to 0, there are fewer connections between the node’s contacts. Hierarchy measures the extent to which the aggregate constraint on the node is concentrated in a single contact. A hierarchy value closer to 0 indicates that the constraint is the same for the node’s relationship with each neighbor, whereas a value closer to 1 indicates that all constraints are concentrated in a single contact. The spanner tends to have higher values of effective size, efficiency, and hierarchy, and lower values of constraint [67]. We used Ucinet [68] to compute the above indices for each node in the global user network.

#### User Coding Based on Contribution to the Echo Chamber

To uncover the mechanisms of intra- and intergroup communication among holders of different interests and viewpoints, we conceptualized two types of social mediators. One was the “echoer,” who only initiated interactions with peers whose interests and viewpoints were highly homogenous, thereby contributing to the formation and even consolidation of echo chambers [45]. The other was the “bridger,” who tended to initiate intergroup dialog across areas of interest and heterogeneous viewpoints, aiming to break down echo chamber barriers [45]. To be specific, for a topic-based echo chamber, if a user only created, retweeted, or commented on tweets of the same topic, the user was considered to be an echoer; otherwise, they were considered to be a bridger. For an attitude-based echo chamber, if a user only created, retweeted, or commented on tweets from users who had the same attitude, the user was classified as an echoer; otherwise, they were classified as a bridger.

## Results

### Descriptive Statistics

**Figure 2:**
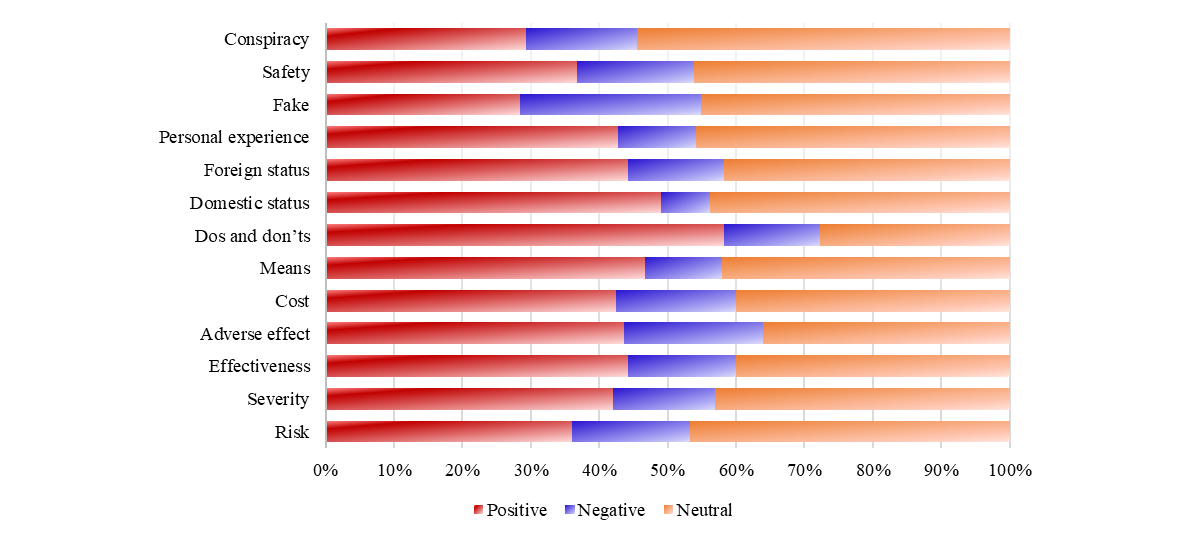
The distribution of attitudes expressed in tweets (original tweets, retweets, comments) about different topics.

Tweets about domestic, foreign status, and conspiracy accounted for 24.46% (n=29,653), 20.40% (n=24,734), and 16.46% (n=19,955) of total tweets (N=121,243), respectively. Overall, 42.51% (51,544/121,243) of tweets expressed a positive attitude toward vaccines and 13.12% (15,907/121,243) of tweets held a negative attitude. Figure 2 shows that discussions about domestic status were the least controversial, whereas discussions related to counterfeit vaccines and fraudulent information were the most divisive.

### Echo Chamber Effect in Networks

**Figure 3:**
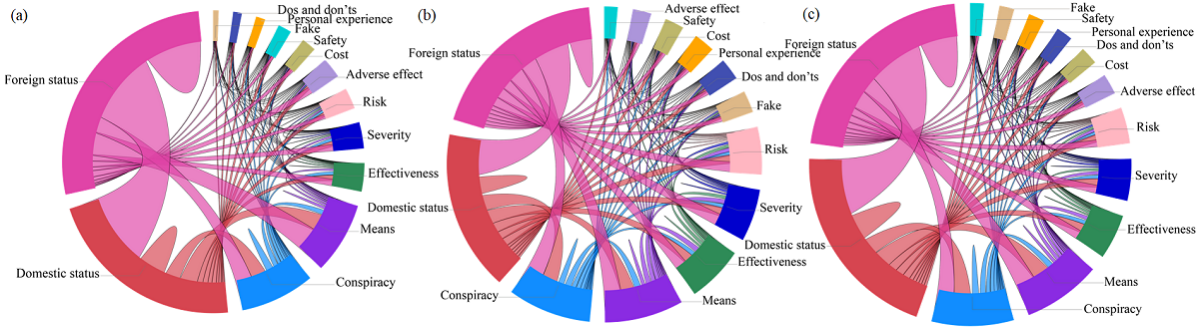
Chord diagram representation of the retweet information network (a), comment information network (b), and global information network (c) colored by topic.

Retweet, comment, and global information networks were all sparse, with a density of 0.003, 0.003, and 0.0002, respectively. In Figure 3, the outer ring of 13 different colors represents a collection of 13 different topics of original tweets, the arc length represents the total connection volume for all of the original tweets belonging to this topic, and the inner colored connecting bands indicate the flow direction and magnitude of the data relationship. The top four topics that interacted most frequently with others were “Foreign status,” “Domestic status,” “Conspiracy,” and “Means.” “Foreign status” was often retweeted by users with topics such as “Domestic status,” “Conspiracy,” and “Means” at the same time, along with “Effectiveness, “Severity,” and “Risk.” The assortativity coefficients of retweet, comment, and global information networks were 0.060, 0.022, and 0.048, respectively, indicating low topic-based homogeneity and that the retweet information network displayed more obvious homogeneity compared with the comment information network.

**Figure 4:**
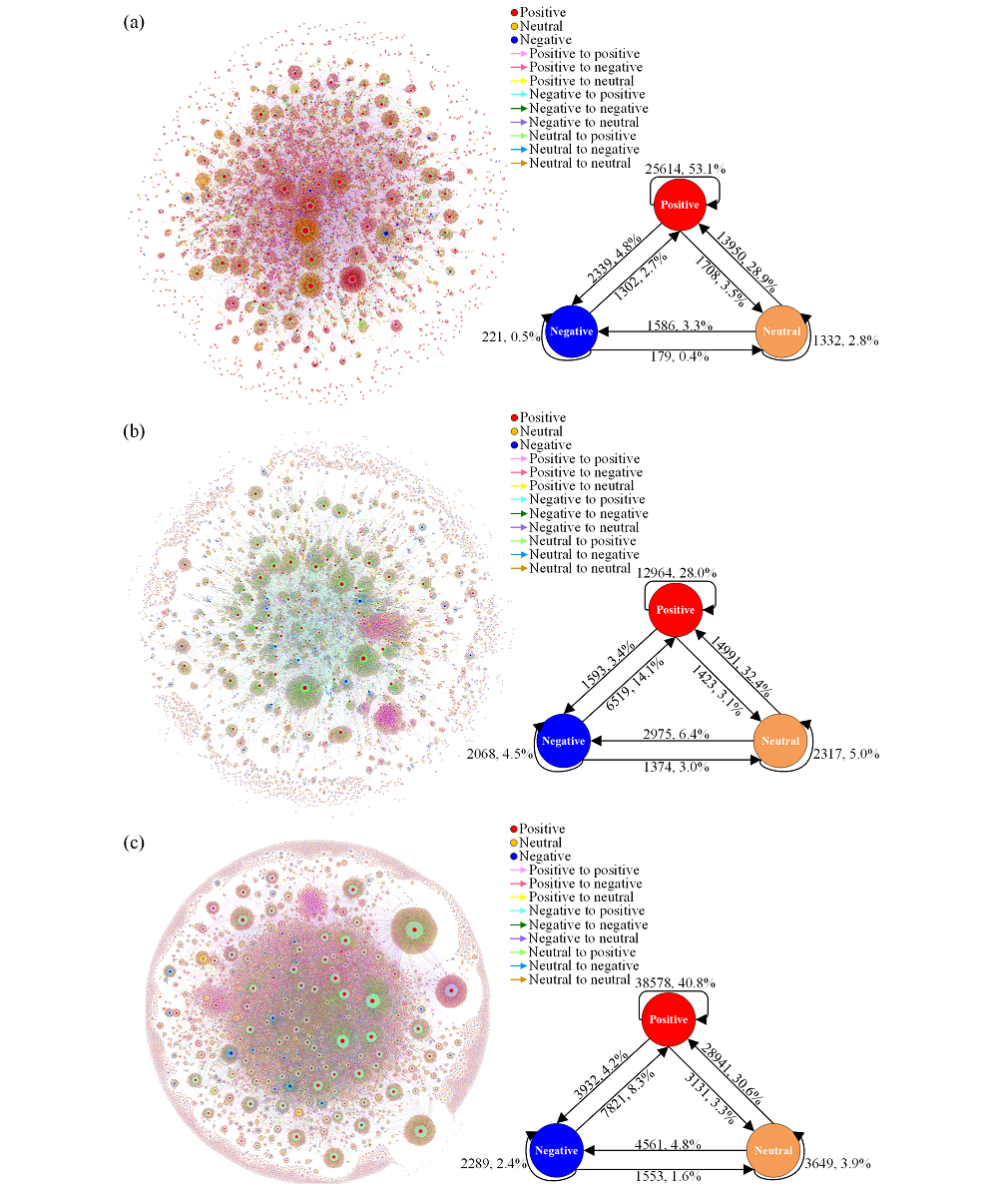
Communication flow network of users in the (a) retweet user network (b) comment user network, and (c) global user network. The size and color of each node represent its in-degree and user’s attitude (red=“positive”, blue=“negative”, orange=“neutral”), respectively. The color of the edge is explained in the corresponding legends in the figure.

Retweet, comment, and global user networks were also sparse, with densities lower than those of information networks. Compared with those of the retweet user network (0.003, 0.0003, 0.011), the comment user network had a higher clustering coefficient, transitivity, and reciprocity (0.007, 0.055, 0.025), indicating that the network built on comment relationships was more cohesive and stable, where users were closely connected and relatively stable [69], while retweeting was mostly used for a one-way flow of information [70]. As shown in Figure 4, in the three user networks, clusters brought together people who were confident about vaccines and people with uncertainty [71]. The more common edges were found between users holding a positive attitude and between users with a neutral attitude to users with a positive attitude. Users with a clear attitude hardly retweeted posts from users without a determined attitude. Compared with the retweet user network, the tendency of users with a negative/neutral attitude to comment on posts of other users with the same attitude was more obvious, whereas this tendency was less obvious for users with a positive attitude. The assortativity coefficients of the retweet, comment, and global user networks were 0.031, 0.042, and 0.055, respectively, indicating low attitude-based homogeneity and that the comment user network displayed more obvious homogeneity compared with the retweet user network.

### Relationships Between User Behavior, Network Position, and Role in the Echo Chamber

**Figure 5:**
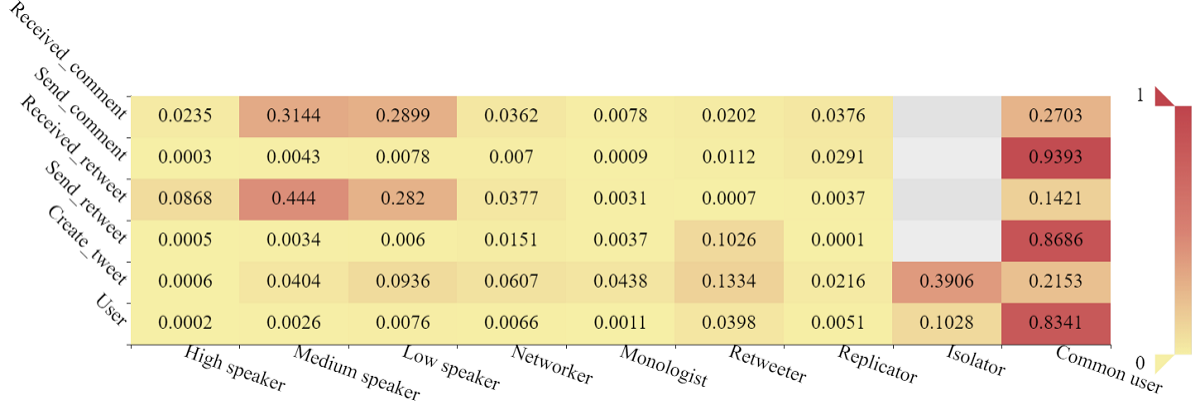
Percentage of user categories based on their behavior. No automatics were detected in the data set.

As shown in Figure 5, most users were coded as “common user,” sending 86.9% of retweets and 93.9% of comments. Only 1.0% of users were speakers, but receiving 81.3% of retweets and 62.8% of comments. Most of the original tweets were created by isolators. Retweeters not only often created tweets but also frequently retweeted others’ tweets.

**Figure 6:**
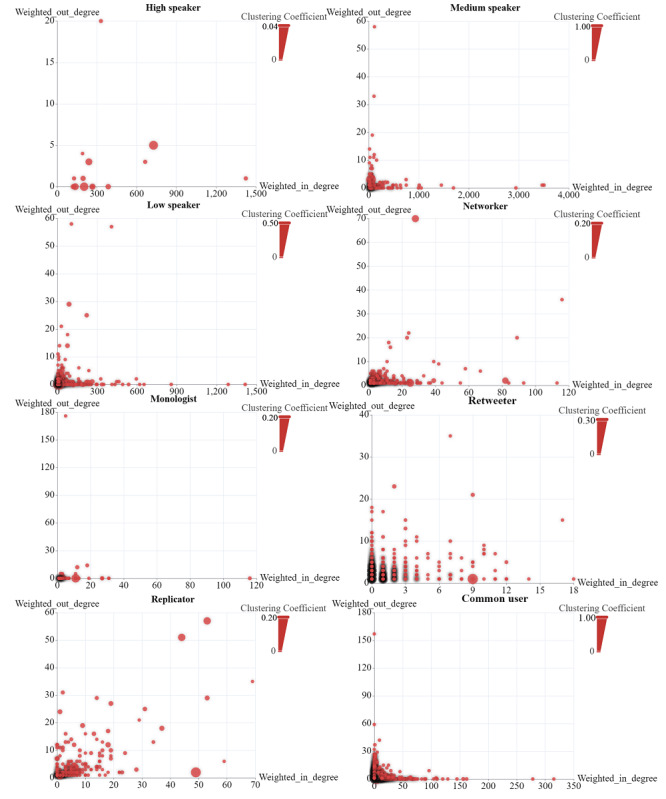
The distribution of users’ weighted in-degree centrality, weighted out-degree centrality, and local clustering coefficient (the size of the circle). The depth of the shadow represents the number of users with corresponding centrality and clustering coefficients.

As shown in Figure 6, with respect to the weighted degree, most of the speakers’ weighted in-degree centrality was much higher than their weighted out-degree centrality. A different situation was found for retweeters. Speakers had a relatively higher average clustering coefficient (high speakers: 0.012, medium speakers: 0.013, low speakers: 0.007), whereas the average clustering coefficient of retweeters (0.0002) was the lowest among users (apart from isolators). Compared with that of retweeters, replicators had a higher average clustering coefficient (0.004), with in-degrees and out-degrees of similar size.

**Figure 7:**
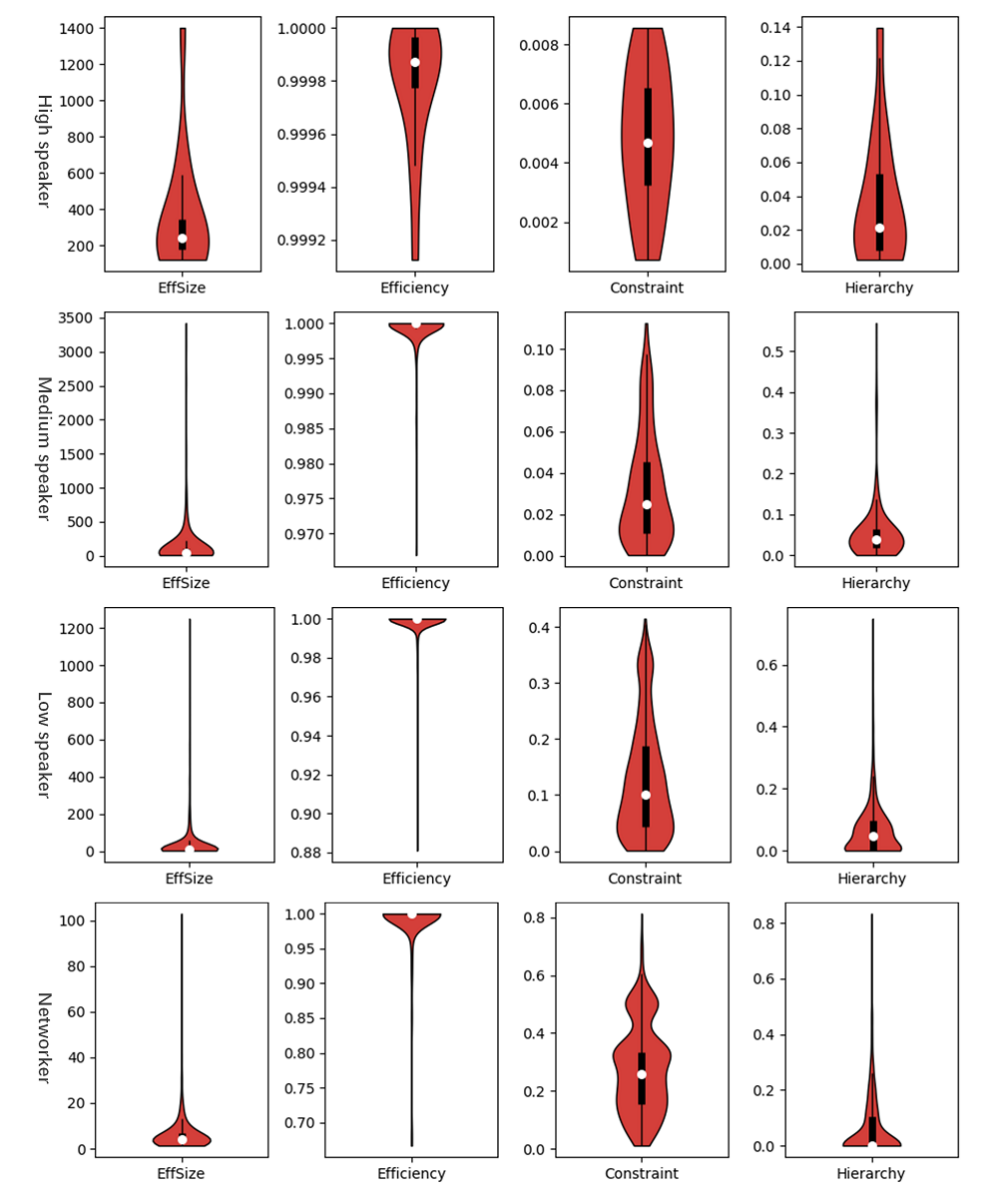
Distribution of users’ structural hole indices (speakers and networks). The white dot, and upper and lower lines of the thick black line represent the index’s median, third quantile, and first quantile, respectively. The width of the red shadow represents the percentage of specific-category users whose index took on that value.

**Figure 8:**
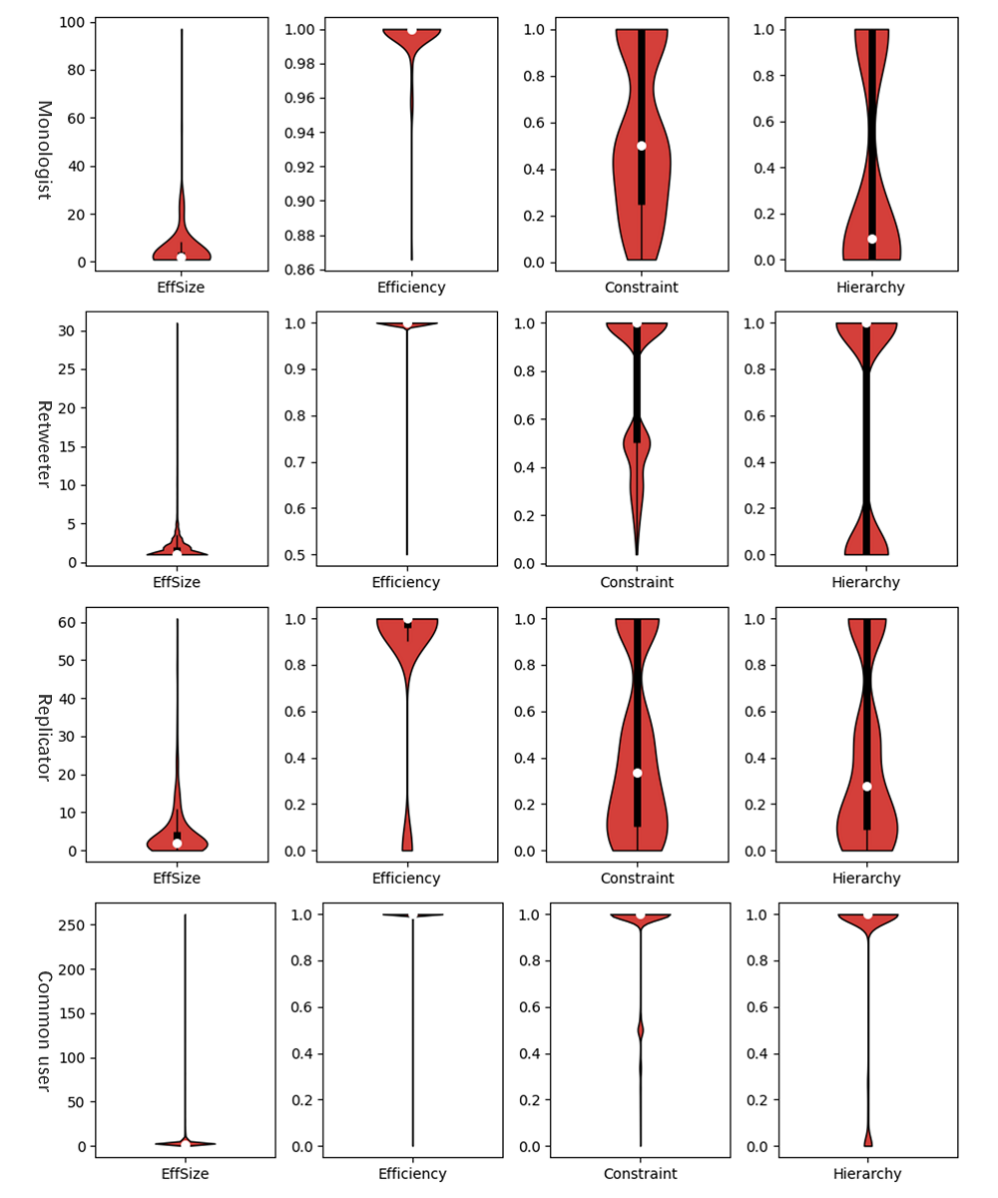
Distribution of users’ structural hole indices (monologists, retweeters, replicators, and common users). The white dot, and upper and lower lines of the thick black line represent the index’s median, third quantile, and first quantile, respectively. The width of the red shadow represents the percentage of specific-category users whose index took on that value.

Figure 7 and Figure 8 show that influentials had more obvious structural hole properties than broadcasters. Among influentials, speakers had a higher effective size, efficiency, and lower constraint than networkers. High speakers performed in the same manner but with a much greater effect. However, some networkers had higher hierarchy than speakers, which indicated that a networker’s constraint was more concentrated on this actor and was more important. Among broadcasters, half of the monologists’ constraint values were lower than 0.500 and half of them had hierarchy values lower than 0.092. Although most of the replicators’ constraint values were lower than 0.333, they largely showed hierarchy values lower than 0.278. Compared with that of replicators, retweeters’ constraint was more concentrated in a single contact.

**Figure 9:**
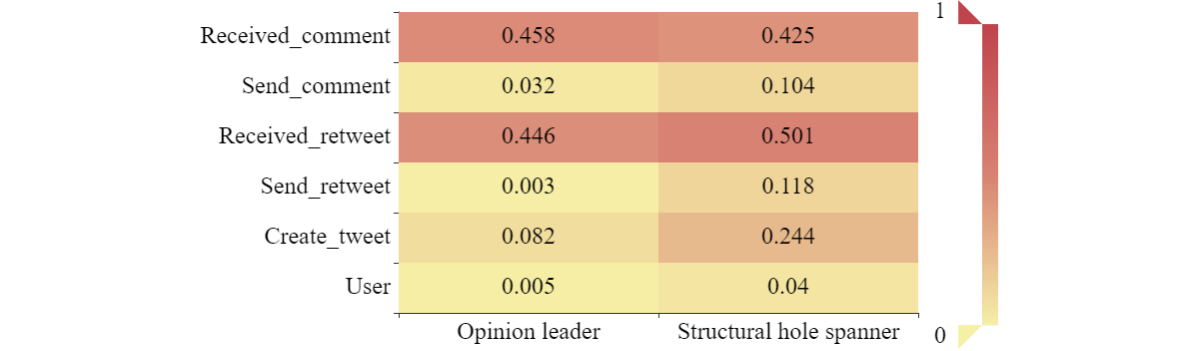
Percentages of tweets from key users.

Given the massive network size, we considered the top 5% of users in weighted in-degree centrality and local clustering coefficient as opinion leaders (n=386, 0.5% of all users), and the other users in the bottom 5% in constraint were considered as structural hole spanners (n=3123, 4.0% of all users). These two types of users were considered key users. As shown in Figure 9, opinion leaders were responsible for 38.4% of all of the information flow, while structural hole spanners were responsible for 50.2% of the information flow. Compared with the former, the latter tended to create, retweet, and comment on more tweets.

**Figure 10:**
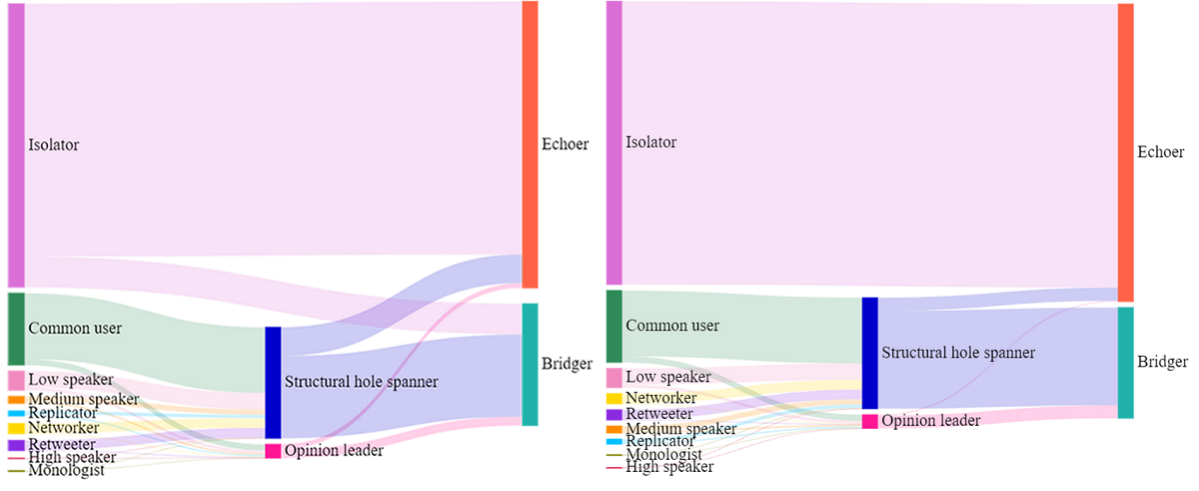
Composition of user categories in opinion leaders/structural hole spanners and their role in the topic-based echo chamber (left) and attitude-based echo chamber (right).

As shown in Figure 10, common users, speakers, replicators, and networkers accounted for 44.0%, 36.3%, 10.6%, and 4.1% of opinion leaders, respectively. Common users, speakers, networkers, and retweeters accounted for 59.3%, 19.7%, 8.7%, and 8.7% of structural hole spanners, respectively. The *χ*^2^ tests showed a significant difference in the distribution of categories of users between opinion leaders and structural hole spanners (*χ*^2^_7_=184.650, *P*<.001). Posthoc testing further showed that speakers, replicators, and monologists tended to be opinion leaders, whereas common users, retweeters, and networkers tended to be structural hole spanners.

Isolators did not become opinion leaders or structural hole spanners, whereas 89.2% of isolaters were topic-based echoers and all of them were attitude-based echoers. The results of *χ*^2^ tests showed that the proportion of structural hole spanners acting as topic-based bridgers (74.2%) was significantly higher than that of opinion leaders (64.2%) (*χ*^2^_1_=17.148, *P*<.001). The opposite result (*χ*^2^_1_=13.193, *P*<.001) was found when considering attitude-based bridgers (structural hole spanners: 88.1%; opinion leaders: 94.3%). Hence, compared with being echoers, both opinion leaders and structural hole spanners tended to act as bridgers. Structural hole spanners were more likely to become bridgers than opinion leaders in topic-based echo chambers, whereas structural hole spanners were less likely to become bridgers than opinion leaders in attitude-based echo chambers.

To address RQ4, the support and the confidence of the rule were calculated. As shown in Table 4, RQ4a was declined, whereas RQ4b was supported, with 62.8% of all key users acting as both topic-based and attitude-based bridgers. Approximately 85.9% of topic-based bridgers also acted as attitude-based bridgers. Specifically, 60.6% of opinion leaders (32.9% government accounts, 30.3% We-media, 19.2% traditional media) and 63.0% of structural hole spanners (39.4% common personnel, 28.4% We-media, 17.3% traditional media) acted as both topic-based and attitude-based bridgers.

**Table 4:** Support and confidence of research question 4 (RQ4).

User category			Number of users		Number of users as topic-based echoers/bridgers		Number of users as both topic-based and attitude-based echoers/bridgers		Support^a^		Confidence^b^
**RQ4a^c^**											
	Opinion leader	386		138		8		0.021		0.058	
	Structural hole spanner	3123		807		23		0.007		0.029	
	Total	3509		945		31		0.009		0.033	
**RQ4b^d^**											
	Opinion leader	386		248		234		0.606		0.944	
	Structural hole spanner	3123		2316		1968		0.630		0.850	
	Total	3509		2564		2202		0.628		0.859	

^a^Support equals the number of users as both topic-based and attitude-based echoers (RQ4a)/bridgers (RQ4b) divided by the total number of users.

^b^Confidence equals the number of users as both topic-based and attitude-based echoers (RQ4a)/bridgers (RQ4b) divided by the number of users as topic-based echoers/bridgers.

^c^RQ4a: Do key users acting as echoers in topic dissemination tend to play the same role in attitude interaction?

^d^RQ4b**:** Do key users acting as bridgers in topic dissemination tend to play the same role in attitude interaction?

## Discussion

### Echo Chamber Effect in Online Vaccine Communication

Users showed an overall low echo chamber effect in vaccine-related topic selection and they tended to comment on more diverse topics than retweeting them. Discussions about the status of vaccine development, and vaccination at home and abroad, mostly mixed with conspiracy, largely caught users’ attention [72]. The risk of contracting COVID-19 and the serious consequences of refusing to be vaccinated were cocommented with claims of vaccine effectiveness.

In contrast to the findings of Mønsted and Lehmann [5] and Schmidt et al [6], users showed a low echo chamber effect in attitude interaction. Because the COVID-19 vaccine represents a medical innovation directly related to the safety of human life, a rational public, threatened by the public health emergency, was less bound by herd mentality [73]. As the dominant opinion, a positive attitude appealed to following of a neutral crowd, which helped to weaken the echo chamber. These findings are inconsistent with those of Rathje et al [19], possibly because national cultural backgrounds influence the cognition, decision-making, and interactive behavior of people belonging to different parties in the United States and United Kingdom. In addition, in contrast to the findings of Tsai et al [45], we found that the overall homophily was more obvious in commenting than in retweeting. Specifically, users approving vaccines showed a more significant tendency to interact with like-minded neighbors by retweeting than by commenting [43], while users against vaccines or with a neutral attitude acted more significantly by commenting than by retweeting, which suggested that the commenting mechanism might serve as an “anti-spiral of silence” to compete with a “silence spiral” in retweeting to form the global opinion climate [74]. Retweeting amplifies the visibility of individuals’ opinions [75], influenced by selective psychology, and opinions contrary to mainstream opinions are silenced. While the commenting network was more modularized and cohesive, users were under greater pressure from within their own communities.

### Users’ Behavior Patterns Contributing to Their Network Positions

The most common behaviors were helpful in spreading information (high percentages of common users and retweeters), while few users frequently participated in two-way dialogs (low percentage of replicators) [62]. Speakers were relatively scarce, but they created content provoking responses of others, which contributed to their popularity in the network, so as to be regarded as information centers within their communities. Networkers who demonstrated a balance between creating content, sharing content, and being retransmitted were more likely to fill structural holes to link otherwise less-connected communities. The commenting mechanism offered more chances to create cohesive communities, and hence nominate replicators in each cluster as opinion leaders, while the opposite situation was found for the retweeting mechanism and retweeters.

Consistent with Yang et al [14], opinion leaders and structural hole spanners tended to have a stronger influence than ordinary users. These two jointly played an important role in making the information propagate over a wider scale; the former affected their entire communities of the network, while the latter connecting to different communities affected the entire network [76]. Specifically, spanners initiated interactions proactively [77].

### Users With Different Network Positions Function in Echo Chamber Formation and Disintegration

Tan et al [66] found that degree centrality and structural holes were complementary at enhancing an organization’s innovation performance in low-density networks. Similarly, we found that both opinion leaders and structural hole spanners played a positive role in breaking the echo chamber for topic dissemination and attitude contagion about COVID-19 vaccines. Opinion leaders insulated others against rather than exacerbated the echo chamber [35,36], which contradicts with the findings of Cossard et al [17]. As gatekeepers, because of social pressure and social support based in part on interpersonal trust [78], they were responsible for filtering, curating, and disseminating information they deemed relevant to their social circle to prevent their followers from being trapped in echo chambers. Structural hole spanners diffused information from one group to another, negotiated and synthesized different topics and standpoints, and promoted cooperation in diverse knowledge and ideological fields [79]. An et al [80] found that the same topic could breed multiple emotions and stakeholders with high topic influence that might not necessarily have high sentiment influence, which, to some extent, explained why users as topic-based echoers might not necessarily act as attitude-based echoers, while users as topic-based bridgers also tended to act as attitude-based bridgers in this study. Aware of the negative impact of echo chambers on crisis management and vaccine promotion, despite different cultural backgrounds, government and We-media positively promoted heterogeneity, and the traditional media’s agenda-setting power was also evident in both topic and opinion spread [29]. Moreover, Wagner and Reifegerste [81] declared that although isolators were rarely found in their interviews, since participants reported communicating about pandemic-related media coverage “with basically everyone,” some participants might turn into isolators during the trajectory of the pandemic. Our findings certainly confirmed this prediction. We found many isolators, which meant that they did not contribute to increasing the scale of information dissemination, which is distinct from the phenomenon noted in disaster-information diffusion [62]. However, the isolators were potential echoers by creating homogenized information and constantly reiterating a single point of view. Although no other users interacted with them, the words of isolators might invisibly reinforce the thoughts of others who saw or read their tweets.

### Theoretical Contributions

The main theoretical contributions of this study are as follows. First, echo chambers in vaccine debates during a crisis differ from those related to general social issues. This study not only examined the echo chamber effect in different information-dissemination dimensions (topic, attitude) and based on different interactive mechanisms (retweeting, commenting), but also dug out the reasons for a low echo chamber effect from the perspective of the relationship of users’ network location and their function in preventing or breaking echo chambers. This offers a powerful complement to existing research focusing on echo chambers’ form, degree, formation, and depolarization.

Second, we focused on two types of key users, namely opinion leaders and structural hole spanners, and characterized their behavioral patterns, which could be a supplement for feature engineering of these key users’ detection or prediction. In addition, referring to the bonding and bridging relationship of social capital, this study proposes two new types of social mediators, namely echoers and bridgers, to quantify key users’ impact on echo chambers, thereby enriching the application scope of social capital theories. Hence, users could be classified based on their behavior, network location, impact on echo chambers, and stakeholder theory [63], offering insights for the construction of user portraits.

Third, previous studies about online key users either focused only on their antecedents (factors contributing to individuals occupying a central location/filling a structural hole [77,82,83]) or only on outcome variables (such as the impact of their locations on knowledge management and innovation performance [84,85], information diffusion [14,76], and emotion contagion [86]). This work linked key users’ antecedents and outcomes at the same time, which could be used to explore hidden behavioral paradigms.

Fourth, we analyzed the relationship of users’ roles in topic-based and attitude-based echo chambers, providing a new research perspective for the dissemination pattern of topic and sentiment.

Finally, most previous studies excluded users who did not interact with others in the data preprocessing step, ignoring their large-scale presence and potential influence on public opinion evolution. This study is thus the first to explore the impact of such users on echo chambers, which could offer a reference for further research about isolators.

### Practical Implications

First, although a low echo chamber effect existed in users’ selection of topics about vaccines, users tended to focus on some specific topics, namely the status of vaccine development, vaccination at home and abroad, and conspiracies. Health medical and public opinion managers should be aware of the emergence of echo chambers centered on these topics, which might damage international cooperation for vaccinations and epidemic control [87].

Second, users with neutral attitudes toward vaccines were easily influenced by others with determined standpoints. The interaction between opposing viewpoints remained limited. Managers should invite online opinion leaders and structural hole spanners who act as bridgers to offer multiple aspects of vaccine knowledge to correct opponents’ misunderstanding and improve their health literacy. At the same time, although provaccine sentiment, as the mainstream opinion, was largely spread and echoed in retweeting, managers should monitor the evolution of other opinions in commenting to prevent the wrong view from turning defeat into victory.

Third, echo chambers have been a major concern of the government, traditional media, and We-media. To obtain better effectiveness, these stakeholders should try to become opinion leaders or structural hole spanners according to their aims by adjusting their own usage behavior on social media. Our results showed that, compared with opinion leaders, structural hole spanners performed better in diffusing diversified topics, whereas opinion leaders performed better in bridging heterogeneous views.

Finally, online isolators should not be ignored. Although these users were reluctant to interact with others and did not receive any feedback from others, they showed interest in creating messages. They were also immersed in personal echo chambers. Managers should take specific measures to break these isolators’ echo chambers.

### Limitations

First, we simply divided users into two categories, namely echoers and bridgers, according to the rule as to whether the user spread more than one topic or interacted with cross-cutting neighbors, rather than quantifying the extent to which they acted as echoers/bridgers using continuous values. Further exploration is therefore warranted. Second, we did not manually find bot accounts in our data set, which was part of the strategy of Villodre and Criado [62] in their study on Twitter data. To date, no tool has been developed for robot account identification for Weibo. In future research, it will be important to develop automated detection algorithms for larger-scale data [88]. Bot accounts could be classified based on their behaviors such as posting repeatedly to appeal for attention or posting maliciously to damage credibility [89], which might have different impacts on echo chambers. Finally, our data were limited to the early stage of vaccine promotion, and we did not consider the impact of subsequent virus variants on public perceptions of vaccines. Updated data should be supplemented in follow-up studies. Moreover, this research should be extended to other social media platforms (eg, Zhihu), users with higher information literacy [90], and in discussions about different controversial social issues to evaluate the consistency or differences from our results.

### Conclusions

By adopting network analysis, this study evaluated and compared the echo chamber effect in users’ topic selection and attitude interaction based on different social media mechanisms (retweeting, commenting) in the vaccine debate during the public health emergency of COVID-19. We further used statistical and visual analyses to characterize behavioral patterns of key users (opinion leaders, structural hole spanners), and explored their function in avoiding/breaking or preventing/strengthening topic-based and attitude-based echo chambers. These findings could provide meaningful inspiration for health medical and public opinion managers to break online echo chambers and eliminate vaccine hesitancy.

## Abbreviations

RQ: research question
